# A novel RF-CEEMD-LSTM model for predicting water pollution

**DOI:** 10.1038/s41598-023-48409-6

**Published:** 2023-11-28

**Authors:** Jinlou Ruan, Yang Cui, Yuchen Song, Yawei Mao

**Affiliations:** Henan Provincial Communications Planning and Design Institute Co., Ltd, Zhengzhou, 450000 People’s Republic of China

**Keywords:** Environmental sciences, Hydrology

## Abstract

Accurate water pollution prediction is an important basis for water environment prevention and control. The uncertainty of input variables and the nonstationary and nonlinear characteristics of water pollution series hinder the accuracy and reliability of water pollution prediction. This study proposed a novel water pollution prediction model (RF-CEEMD-LSTM) to improve the performance of water pollution prediction by combining advantages of the random forest (RF) and Long short-term memory (LSTM) models and Complementary ensemble empirical mode decomposition (CEEMD). The experimental results based on measured data show that the proposed RF-CEEMD-LSTM model can accurately predict water pollution trends, with a mean ab-solute percentage error (MAPE) of less than 8%. The RMSE of the RF-CEEMD-LSTM model is reduced by 62.6%, 39.9%, and 15.5% compared to those of the LSTM, RF-LSTM, and CEEMD-LSTM models, respectively, proving that the proposed method has good advantages in predicting non-linear and nonstationary water pollution sequences. The driving force analysis results showed that TN has the most significant impact on water pollution prediction. The research results could provide references for identifying and explaining water pollution variables and improving water pollution prediction method.

## Introduction

With the rapid advancement of industrialization and urbanization, water pollution problems have become increasingly serious in China^1^. Accurate and reliable water environment prediction models can provide real-time water pollution concentration change information, enabling health risks to be avoided in a timely manner and providing an intuitive reference for environmental protection departments. Water environments are nonlinear, nonstationary, and noisy systems, making water environment prediction difficult^2^. How to deeply explore and extract the laws contained in the concentration series of water pollutants and accurately predict the change trends of water pollutants in the future has become a difficult and urgent problem to solve.

Numerical models and data-driven models are mainly used to predict the concentration of pollutants in water environments^3^. Numerical models can simulate the development of pollutants and predict the quality of a water environment^4^. Fu et al.^5^ analyzed the characteristics and challenges of existing water quality models and found that in terms of model parameter calibration, although a comprehensive calibration plan has been established^6^, there are still difficulties in combining the model with on-site or laboratory observation results. Due to limited data, high-dimensional models, and overreliance, the numerical models may not be able to accurately capture all functional properties of the water quality variables of interest, resulting in significant difficulties in calibrating the model^5^. This uncertainty of model parameters makes it difficult for the constructed model to accurately simulate the potential relationship between input and output variables. Data-driven models do not require complex fluid dynamics theories and complex processes^7^. They can effectively explore the potential relationship between input variables and target variables by utilizing a large amount of historical monitoring data, and have superior applicability compared to numerical models^8^. Among them, artificial neural networks (ANNs) models are widely used in pollutant prediction due to their excellent ability to learn linear and nonlinear information from historical data^9^. Rustam et al.^10^ used ANN in water quality and water quantity predictions, and the results showed that the accuracy of water quality prediction was 0.96, verifying the feasibility of artificial neural networks in water quality prediction. Najwa Mohd Rizal et al.^11^ compared the performance of regression models, support vector machines, and ANNs in water quality prediction, and the results demonstrated that the ANN model was superior to the other models. Although these data-driven models can achieve good predictive performance, a single machine learning model is susceptible to overfitting and often fall into local optima^12^.

In response to this problem, scholars have attempted to use combination forecasting models to predict water pollution. The composite model skillfully combines multiple models, aiming to solve the defects of a single model^13^. Common combination models include the residual processing model^14^, weight combination model^15^, and data decomposition model^16^. The residual processing model improves the prediction accuracy by processing the residuals of the prediction results, but it does not change the scope of application of a single model^17^. Therefore, it has great limitations in dealing with highly noisy, nonstationary and nonlinear systems. The weight combination model improves the accuracy and stability by assigning appropriate weights to each submodel to offset the residual prediction results. One weight combination model is often only applicable to specific data^18^. Water pollution data have significant nonlinear and nonstationary characteristics, leading to the phenomenon of high training accuracy but low verification accuracy when using weighted combination models to predict water pollution. Recent studies have shown that the empirical mode decomposition (EMD) is an effective data preprocessing method, which can decompose the original time series data into multiple subsequences with different frequencies, enabling the regular information contained in the data to be fully recognized and extracted, and is widely used in sequence prediction^16^. Hybrid models coupling EMD and machine learning tools have been commonly used in the water environment fields^19^. To improve the accuracy of prediction methods, Zhang et al.^20^ used empirical mode decomposition (EMD) to preprocess the data and then used LSTM to predict water quality indicators, and found the performance of the hybrid models was superior to that of the single model. Due to the strong dependence of EMD on signal frequency, amplitude, and their differences, mode mixing often occurs during data decomposition^21^. The ensemble EMD (EEMD) method is an improved form of EMD that can overcome the modal mixing^22^. Eze et al.^21^ developed a new combined prediction method using EEMD and LSTM neural networks to improve the accuracy of water quality parameter prediction, and found that the performance of the hybrid model is superior to similar water quality parameter prediction models.

One major concern of EEMD is that the introduction of noise assisted analysis increases computational complexity and time consumption. Moreover, the introduction of noise has a certain degree of damage on the original signal, leading to potential uncertainty in the decomposition results^23^. The complementary ensemble empirical mode decomposition (CEEMD) proposed by Yeh et al.^24^ effectively overcomes these difficulties. CEEMD decomposes nonlinear and nonstationary sequence data into multiple components and residual terms by introducing complementary white noise, reducing the impact of residual noise while allowing outlier data to potentially play positive roles^25^. Nevertheless, for short-term water pollution time series with nonlinear and nonstationary traits, whether or to what degree, the hybrid model coupling CEEMD and deep learning models can improve the prediction accuracy remains unclear.

Selecting predictive variables is crucial in determining the performance and accuracy of the model, and recent research suggests using other techniques to select predictive factors before constructing a water pollution prediction model^19^. However, most data-driven models directly use machine learning methods to predict subsequences, ignoring the impact of feature selection on model performance, resulting in significant deficiencies in the interpretation of water pollution causes. There are also significant shortcomings in the analysis of water pollution characteristics. The effective interpretation of water pollution characteristics is often an important basis for water pollution prevention and control.

Considering the above factors, this study attempts to propose a RF-CEEMD-LSTM model for different water quality indicators by combining the advantages of the RF, LSTM model and CEEMD. First, RF was used to analyze the importance of water quality, meteorology, and air quality indicators to water pollution. Second, CEEMD was used to decompose the water pollution prediction indicators into intrinsic mode function (IMF) components and residual terms. Third, the LSTM algorithm was used to construct a combined water pollution prediction model for different water quality prediction indicator components. Finally, the proposed model was used to predict pondus hydrogenii (PH), ammonia nitrogen (NH3-N), and dissolved oxygen (DO) at the Kangdian and Banqiao stations in the Huaihe River basin. RF-CEEMD-LSTM was compared with other models to verify the effectiveness of the model.

## Materials and methods

### Study area and data

The Huaihe River Basin (111°55′E–121°25′E, 30°55′N–36°36′N) is located in central and eastern China and is the third largest water system in China. Its main stream flows through Hubei, Henan, Anhui, and Jiangsu provinces, with a watershed area of 270,000 km^2^. The average annual temperature in the Huaihe River basin is between 11 and 16 ℃, with the highest and lowest temperatures occurring in July and January, respectively, and the average annual precipitation is 920 mm^26^. Due to the developed economy in the area where the Huaihe River flows, the Huaihe River is heavily affected by artificial intervention, resulting in a low capacity to absorb pollution and relatively serious pollution. Since the 1980s, water pollution accidents in the Huaihe River have occurred frequently, with Zhoukou in Henan being the most serious^27^. The water pollution problem has seriously restricted the development of the economy in the basin. Exploring accurate methods for analyzing and predicting water pollution characteristics is of great significance for utilizing water resources, preventing water pollution incidents, and comprehensively managing the environment.

**Figure 1:**
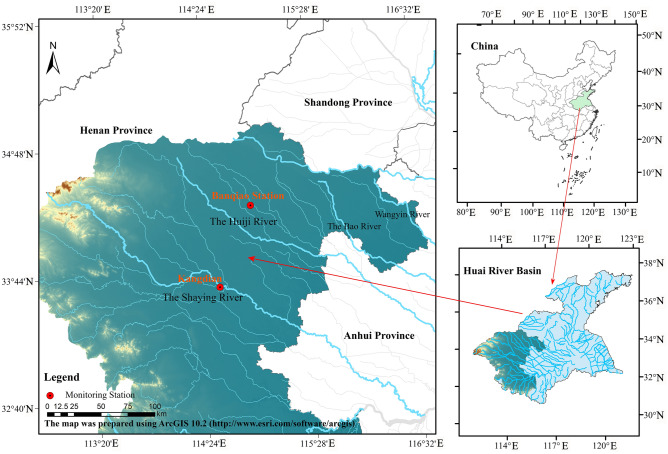
The location of the study area.

The water pollution data, meteorological data, and air quality data of Kangdian station and Banqiao station in the Huaihe River Basin were selected to establish the water pollution prediction model (Fig. 1). The water pollution data are daily monitoring data from January 1, 2021, to December 31, 2022, at the Kangdian and Banqiao stations, these data include pH, water temperature (W-Temp), chemical oxygen demand (COD_Mn_), DO, NH3-N, total nitrogen (TN), total phosphorus (TP), electrical conductivity (EC), and turbidity (Turb). The water pollution data were obtained from the China Environmental Monitoring Station and the hydrological monitoring division of the Huaihe River Hydrology Bureau. The meteorological data, including daily temperature (A-Temp), wind speed (WS), and precipitation (Prep), from January 1, 2021, to December 31, 2022, were obtained from the Resource and Environmental Science and Data Center of the Chinese Academy of Sciences. The air quality data, including daily PM_2.5_, SO_2_, NO_2_, and CO, from January 1, 2021, to December 31, 2022, were obtained from the national air quality real-time release platform.

DO, pH, and NH3-N are important factors that affect the water environment quality and are also the focus of attention in water pollution prevention. The pH value is the negative logarithm of the hydrogen ion concentration in water, reflecting the degree of acid and alkali pollution of a water body. DO reflects the self-purification ability of a water body. A higher DO content indicates a strong self-purification ability of the water body. NH3-N is an important indicator that reflects the nutritional status of water bodies. NH3-N is present in water as free ammonia or ammonium salt and is the main oxygen-consuming pollutant in water bodies. Therefore, a water pollution prediction model was constructed with these three indicators.

### Data filling

Due to the impact of monitoring station maintenance, there are a few missing data points in the sample. Therefore, the sample data were processed into continuous and complete sequence data. Cubic spline interpolation was used to process the missing data. Cubic spline interpolation can effectively overcome the limitations of polynomial interpolation and is widely used in the interpolation solution process. In cubic spline interpolation, the interval [a, b] is divided into n intervals [(a, x_1_), (x_1_, x_2_), …, (x_n-1_, b)], and the cubic spline interpolation results are calculated by solving matrix equations. The detailed mathematical description of cubic spline interpolation can be found in Chand et al.^28^. Twenty-three missing data points were processed using cubic spline interpolation (Eq. 1), accounting for 0.53% of the total sample data. The December 2022 data were selected as the test set, and the data from January 2021 to November 2022 were selected as the training set.

1 $${\text{y = }}a_{i} + b_{i} x + c_{i} x^{2} + d_{i} x^{3} .$$

$a_{i} ,b_{i} ,c_{i} ,d_{i}$ refers to the coefficients that need to be solved in each interval.

### RF for feature selection

Input variables are the key factors that affect the performance of water pollution prediction. Quantifying the impact differences of water pollution indicators can explain the characteristics and causes of water pollution and provide a reference for water pollution prevention and control. But there is no unified paradigm for selecting and quantifying water pollution prediction indicators. In view of the above considerations, a method for selecting water pollution prediction indicators based on the RF algorithm was proposed in this study.

The RF algorithm is an integrated learning method based on decision trees^29^. In the RF algorithm, K training sets are randomly extracted using bootstrap resampling technology, and K decision trees are trained to form the random forest^30^. A RF has the advantages of simple modeling and strong generalizability^31^ and exhibits better performance than that of individual decision trees in many classification analyses^31^ and prediction tasks^30^. RF can also be used as a feature selection method.

In the quantitative analysis of water pollution prediction features using RF algorithms, the out-of-bag (OOB) error was used to measure the features. OOB data refers to data that have not been sampled during the RF resampling process, which can be used to evaluate the performance of decision trees. The RF algorithm analyzes the importance of a feature by perturbing it. If the OOB error of the RF model decreases significantly after perturbing the feature, it indicates that the feature is of high importance. The detailed steps for the quantitative analysis of water pollution prediction features based on the RF algorithm^29^ can be divided into four steps.

1. Randomly extract n data from the initial dataset and generate K new training sets. The data that were not extracted constitute K OOB datasets.
2. For each decision tree in the RF, use the corresponding OOB data to calculate the OOB error ($eo_{k}$).
3. Randomly perturb the characteristics of the OOB datasets $x_{i} ,i = 1,2,...,M$ and calculate the OOB error ($eo_{ki}$).
4. Calculate the feature importance of each feature.
  $k$ refers to the number of decision trees, $eo_{k}$ refers to the OOB error of the kth decision tree, $eo_{ki}$ refers to the OOB error of the decision tree after perturbing the ith feature, and $IM_{i}$ refers to the importance score of the ith feature.

After obtaining the feature importance of each input variable to the predicted variable, the variable perturbation method is used to select the fewest number of inputs that offer the best predictive power and the most interpretation of the water pollution prediction model.

### CEEMD prediction sequences

Due to the significant nonstationarity and uncertainty of a pollutant sequence, it is difficult for a model to accurately capture all the characteristics of the sequence, resulting in poor fitting and prediction performance. Therefore, the CEEMD method was used to decompose water pollution prediction sequences into stable components and residual terms before establishing the prediction model. CEEMD is an improvement of the EMD method^24^. EMD can process sequence data into IMF and residual terms that vary in frequency and are relatively stable. But EMD often exhibits the phenomenon of modal aliasing when decomposing sequence data that contain a large amount of noise^32^. To solve this problem, scholars in signal research have proposed various improved sequence decomposition methods, including EEMD^22^ and CEEMD. CEEMD reduces the phenomenon of modal aliasing and the number of iterations required for decomposition^33^ by adding a white noise pair with opposite signs during the data decomposition process. The decomposition process of the water pollution prediction sequence using CEEMD^24^ was shown in Eqs. (2)–(4).

1. Add a white noise pair $\varepsilon_{i} (n)$, denoting the sign, to a given sequence of pollutant concentrations $x(n),n = 1,2,...,N$ (Eq. 2).
  2 $$\left\{ \begin{gathered} x_{i}^{ + } (n) = x(n) + \varepsilon_{i}^{ + } (n) \hfill \\ x_{i}^{ - } (n) = x(n) + \varepsilon_{i}^{ - } (n) \hfill \\ \end{gathered} \right.$$
2. Use CEEMD to decompose each original water pollution sequence with white noise and obtain m IMF components and one residual component $Res$ (Eq. 3).
  3 $$\left\{ \begin{gathered} x_{i}^{ + } = \sum\limits_{j = 1}^{m} {c_{ij}^{ + } } (n) \hfill \\ x_{i}^{ - } = \sum\limits_{j = 1}^{m} {c_{ij}^{ - } } (n) \hfill \\ \end{gathered} \right.$$
  $c_{ij}^{{}}$ refers to the jth modal component of the i-th sequence after CEEMD.

(3) Calculate the average of all IMF components to obtain the final modal component group $c_{i}^{{}} (t)$ by using Eq. (4).
  4 $$c_{i} (t) = \frac{1}{2m}\sum\limits_{j = 1}^{2m} {c_{ij}^{{}} }$$

### Water pollution prediction model based on RF-CEEMD-LSTM

Based on the feature importance analysis of water pollution indicators and modal decomposition of prediction sequences, the water pollution prediction model was constructed using the LSTM method for each decomposed sequence. LSTM is a very important recurrent neural network (RNN) in deep learning methods^34^. An RNN creates a loop by adding additional weights to the network, making its input dependent not only on the current input but also on previous inputs. RNNs often experience gradient disappearance and explosion in long sequence modeling, resulting in a significant decrease in prediction performance. LSTM effectively solves the long-term dependency problem of RNNs by introducing input gates, forget gates, and output gates to store and update cell states, achieving selective retention of the sequence information^35^.

**Figure 2:**
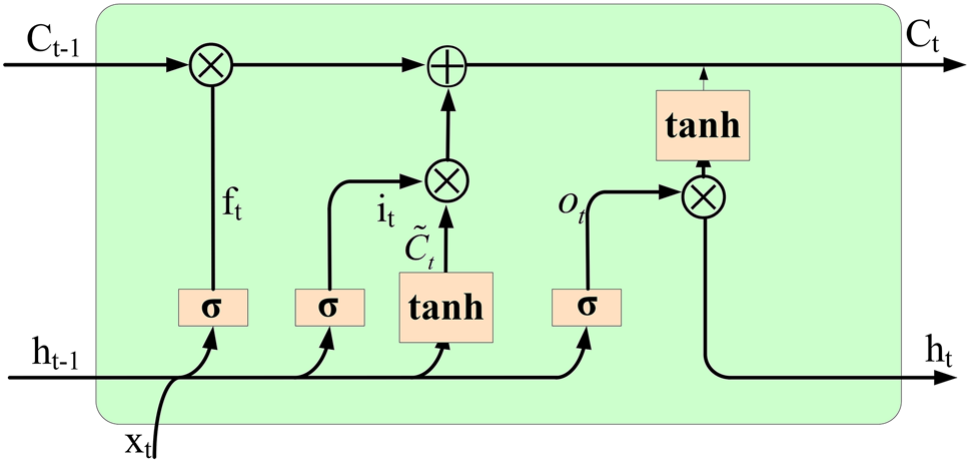
The principle of LSTM.

The structure of LSTM is shown in Fig. 2. The core of LSTM lies in the cell state and the "gate" structure^34^. The cell state can continuously transmit relevant information during sequence processing. The earlier information can be carried to later cells, overcoming the impact of short-term memory. The three "gate" structures pass through σ functions to process data and learn which information to retain or forget during training, overcoming the long-term dependence of information. The main principles of using LSTM^34^ for water pollution prediction are shown in Eqs. (5)–(10).

First, the input gate determines how much new water pollution data information and information output by the previous layer can be transmitted to the cell state.

5 $$i(t) = \sigma [w_{i} \times (h_{t - 1} ,x_{t} ) + b_{i} ]$$

6 $$\tilde{C}_{t} = \tanh [w_{c} \times (h_{t - 1} ,x_{t} ) + b_{c} ]$$

$i(t)$ in Eq. (5) refers to the output value of the input gate, $w_{i}$ and $b_{i}$ refer to the weights and biases of the input gate, respectively, $w_{c}$ and $b_{c}$ refer to the weights and biases of the update cell, respectively, $\sigma$ refers to the activation function (sigmoid), $h_{t - 1}$ refers to the output of the memory cells at time t − 1, $x_{t}$ refers to the input at time t, and $\tilde{C}_{t}$ in Eq. (6) refers to the state of the cell to be updated.

When information from the input gate is passed to the forget gate, the forget gate determines how much real-time data information and information output by the previous layer will be discarded.

7 $$f_{t} = \sigma [w_{f} \times (h_{t - 1} ,x_{t} ) + b_{f} ]$$

$f_{t}$ in Eq. (7) refers to the output of the forget gate and $w_{f}$ and $b_{f}$ refer to the weights and biases of the forget gate, respectively.

The update of the cell state determines the proportion in which past information and instant information are combined and transmitted to the new cell state.

8 $$c_{t} = i_{t} \times \tilde{c}_{t} + f_{t} \times c_{t - 1}$$

$c_{t}$ and $c_{t - 1}$ in Eq. (8) refer to the cell states at time t and time t − 1, respectively.

Finally, the output gate determines how much data information will be output from the cell state. The output data information is used as input to the new round of the model cycle.

9 $$o_{t} = \sigma [w_{o} \times (h_{t - 1} ,x_{t} ) + b_{o} ]$$

10 $$h_{t} = o_{t} \times \tan (c_{t} )$$

$o_{t}$ in Eq. (9) refers to the output value of the output gate, $w_{o}$ and $b_{o}$ refer to the weights and biases of the output gate, and $h_{t}$ in Eq. (10) refers to the output of the memory cells at time t.

The LSTM prediction models were constructed for all prediction sequences decomposed by CEEMD, and all prediction sequences were integrated to generate water pollution prediction results. Parameter optimization is an important step in constructing the LSTM prediction model. The mean square error (MSE) was selected as the loss function, and adaptive moment estimation (Adam) was used as the optimizer in this study. The Adam optimizer combines the advantages of the AdaGrad and RMSProp optimization algorithms, i.e., high computational efficiency and parameter interpretation^36^. The number of neurons, learning rate, iteration times, and sliding window step length of LSTM were optimized by Adam. When the error between the actual value and the predicted value met the accuracy requirements, the model was saved. Based on the characteristics of the data in this study and relevant research results, the allowable error was set to 0.001.

### Schematic diagram of the proposed method

**Figure 3:**
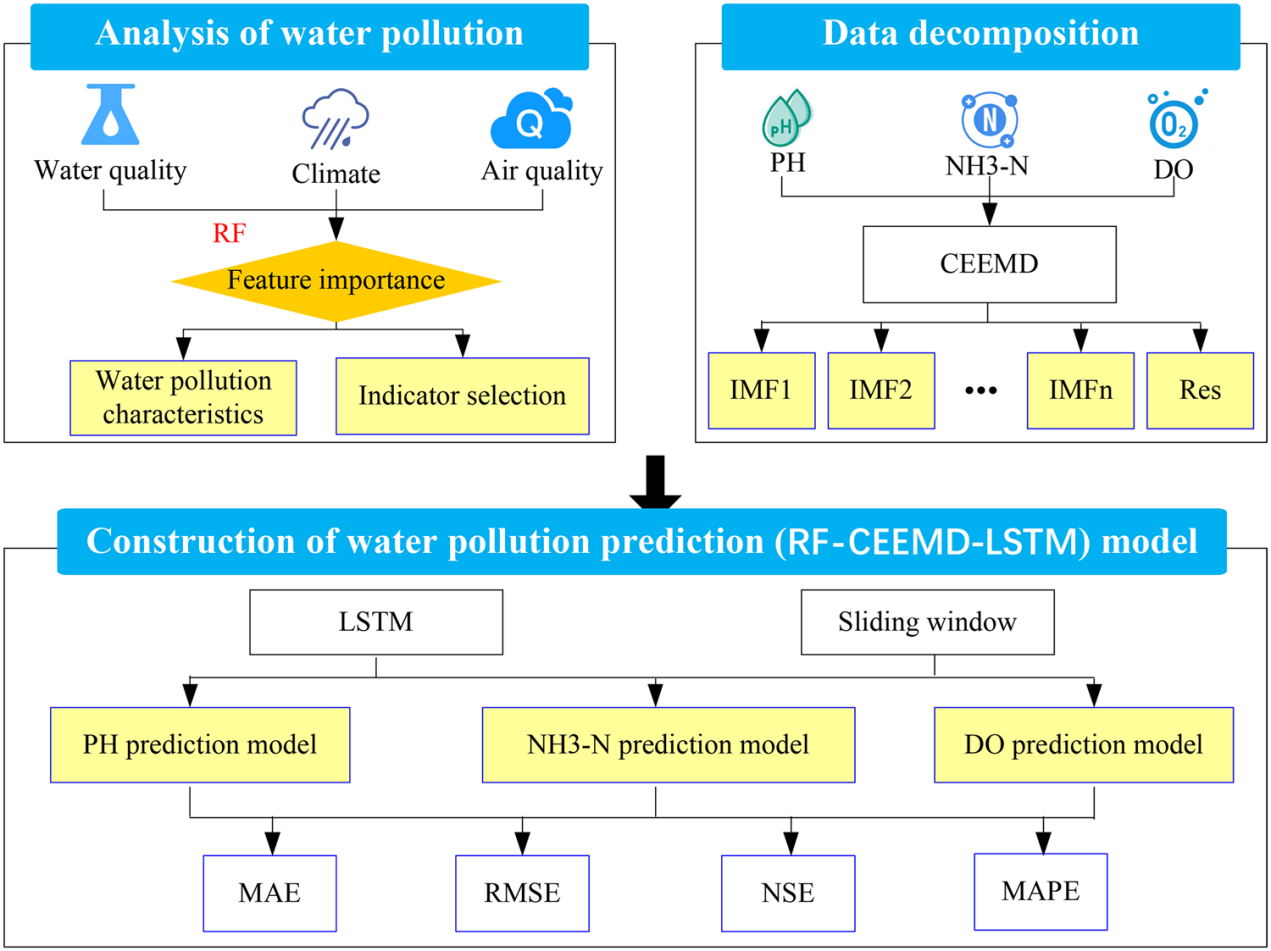
Modeling process of the RF-CEEMD-LSTM model.

The RF algorithm was used for feature importance analysis to quantify the main indicators that affect water pollution prediction sequences in this study. Based on the results of feature importance analysis, an indicator set for different water pollution prediction sequences was proposed. CEEMD was used to reconstruct nonstationary and nonlinear prediction sequences into relatively stable components and residual terms for three types of water pollution prediction sequences from two stations. And LSTM was used to fit and predict trend components and integrate the results of all trend components to obtain the prediction model for different pollution sequences (Fig. 3). To verify the superiority of the proposed model, various other algorithms were used in this study for comparison, including the LSTM, RF-LSTM, and CEEMD-LSTM models.

### Model performance evaluation

To evaluate the performance of the proposed RF-CEEMD-LSTM water pollution prediction model, four statistical indicators were selected to measure the prediction results: Nash–Sutcliffe efficiency (NSE), root mean square error (RMSE), mean absolute percentage error (MAPE), and mean absolute error (MAE). The MAPE (Eq. 13) and MAE (Eq. 14) can reflect the predicted errors in real-world scenarios. The RMSE (Eq. 12) is an evaluation index of the average error and volatility of the predicted results. The MAPE can measure the accuracy of time sequence prediction. The NSE (Eq. 10) can evaluate the fitting ability of the model. The closer the values of MAE, MAPE, and RMSE are to 0 and the closer the value of NSE is to 1, the better the prediction accuracy of the model. The indicator calculation methods are shown in Eqs. (11)–(14).

11 $$NSE = 1 - \frac{{\sum\limits_{i = 1}^{n} {(\hat{y}_{i} - y_{i} )^{2} } }}{{\sum\limits_{i = 1}^{n} {(y_{i} - \overline{y}_{i} )^{2} } }}$$

12 $$RMSE = \sqrt {\frac{{\sum\limits_{i = 1}^{n} {(y_{i} - \hat{y}_{i} )^{2} } }}{N}}$$

13 $$MAPE = \frac{1}{n}\sum\limits_{i = 1}^{n} {|\frac{{y_{i} - \hat{y}_{i} }}{{y_{i} }}|} \times 100\%$$

14 $$MAE = \frac{1}{n}\sum\limits_{i = 1}^{n} {|y{}_{i} - \hat{y}_{i} |}$$

$y_{i}$ is the measured value, $\hat{y}{}_{i}$ is the predicted value of the water pollution, and n is the total number of validation samples.

## Results

### Characteristics of water pollution data

**Figure 4:**
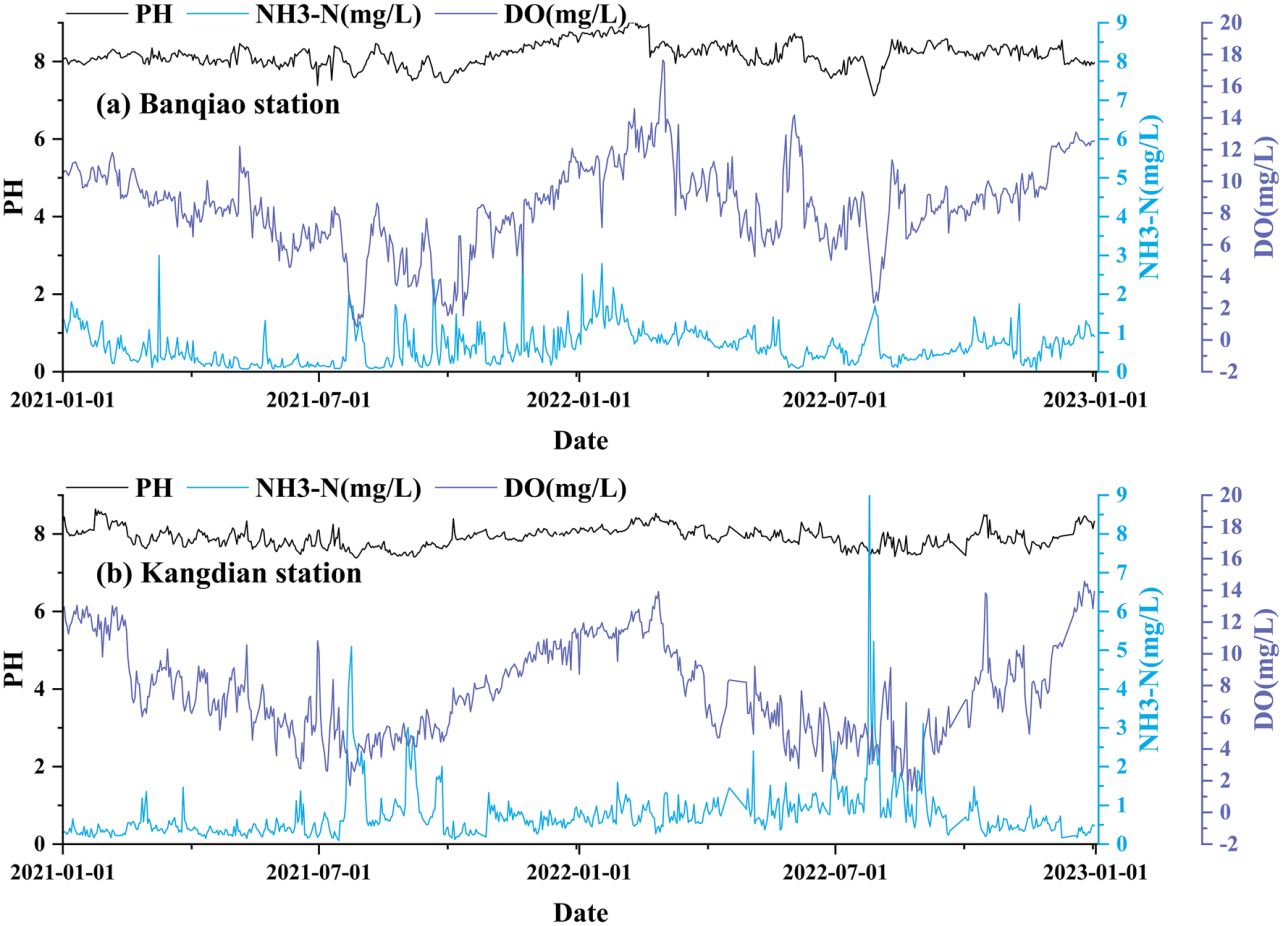
The data characteristics of water pollution prediction indicators (above is the Banqiao Station and below is the Kangdian Station).

A descriptive statistical method was used to analyze the basic characteristics of the water pollution data used in this study to understand the quality and volatility of these data. As shown in Table 1, the missing rates of water pollution data at the two stations were relatively low, 0.37% and 0.69%. The main reason for missing data was that the stations were under maintenance. Cubic spline interpolation was used to process complete missing data in the sample to ensure sequence prediction continuity. Table 1 reflects the overall water pollution at the Kangdian and Banqiao stations. In terms of pH value, the average, maximum, and minimum values of the two stations were between 6 and 9, which conforms to the Class III water quality standard in China^37^. The minimum value of DO (0.83) and maximum value of NH3-H (9.67) at the Kangdian and Banqiao stations were far lower than the Class III water quality standard in China (DO > 5, NH3-H < 5)^37^. These results indicate that there is a certain degree of water pollution in the Huaihe River basin. In addition, Fig. 4 shows that the NH3-H and DO data of the two stations during the flood season (June to September) in the Huaihe River Basin were significantly lower than those during the nonflood season. The main reason is that the flow in the nonflood season is small, resulting in worse water environment quality under the premise of the same pollutant emissions. Therefore, the phenomenon of water pollution in the Huaihe River Basin during the nonflood season is more prominent.

**Table 1:** Descriptive statistics of the water pollution data.

Station	Feature	Mean value	Max value	Min value	Standard deviation	Missing data rate (%)
Kangdian	pH	7.91	8.64	7.38	0.24	0.35
	NH3-N (mg/L)	0.76	9.67	0.66	0.68	0.36
	DO (mg/L)	7.63	14.57	1.31	2.89	0.39
Banqiao	pH	8.17	9	7.11	0.31	0.61
	NH3-N (mg/L)	0.64	3.32	0.025	0.45	0.73
	DO (mg/L)	8.44	17.64	0.83	2.69	0.74

Table 1 and Fig. 4 demonstrate the presence of significant nonlinearity and uncertainty in the water pollution data collected at the Kangdian and Banqiao stations, and there were significant volatility and notable variations among the NH3-H and DO data. The pH data exhibited minimal fluctuation and had the lowest standard deviation among the data collected at the Kangdian and Banqiao stations. The three sets of sample data (pH, NH3-H, and DO) have different data characteristics. Thus, the prediction performance of the combined prediction model can be fully verified.

### Feature selection results based on the RF algorithm

The RF algorithm was used to calculate the importance of water quality indicators, hydrometeorological indicators, and air quality indicators for COD_Mn_, NH3-N, TN, and TP, and the input variable was selected based on the characteristic importance of each indicator. To avoid the contingency of feature importance analysis, the average of 10 feature importance calculations was used as the final importance of each feature. It was confirmed that indicators with a feature importance score exceeding 0.1 have a significant impact on predictive variables^38^. The indicator with importance score exceeding 0.1 was selected as the input variable for the water pollution prediction model in this study.

**Figure 5:**
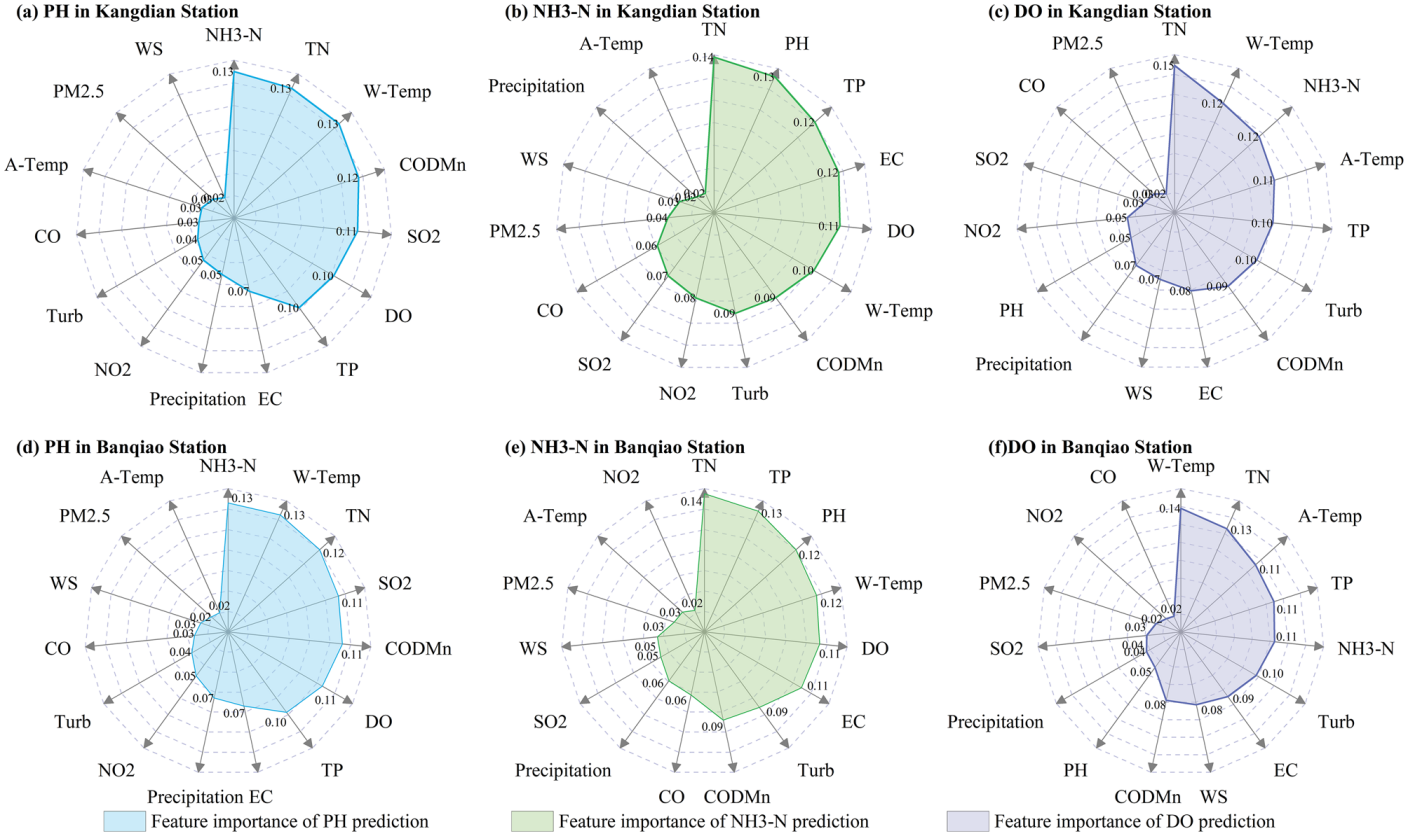
Results of feature importance analysis.

Figure 5 shows the results of the feature importance analysis. At the Kangdian and Banqiao stations, the main factors affecting pH were NH3-N, TN, W-Temp, COD_Mn_, SO_2_, DO, and TP, of which NH3-N had the highest characteristic contribution to pH, indicating that changes in pH are closely related to changes in NH3-N. The results of the importance analysis indicated that SO_2_ in air was also an important factor affecting pH. The main reason is that SO_2_ mainly comes from industrial emissions. The higher the SO_2_ content is, the higher the amount of industrial pollutants discharged. The presence of industrial pollutants in the river significantly affect the pH of the water body. Therefore, pH changes are not only affected by water quality indicators but are also closely related to air quality indicators. Figure 5 also demonstrates that the main factors affecting NH3-N include TN, pH, TP, EC, DO, and W-Temp, of which TN and pH have the greatest impact on NH3-N. The main reason is that TN reflects the total amount of organic and inorganic nitrogen (including NH3-N) in the water body. Therefore, as a component of TN, NH3-N is directly affected by changes in TN. Furthermore, it has become an indisputable fact that water temperature is an important factor affecting DO. The results of the feature importance analysis also indicate that W-Temp and A-Temp are important factors affecting DO. From the results of the feature importance analysis, it was found that TN also had a significant impact on DO. Special attention should be given to changes in TN and temperature in the prediction of DO. Based on the results of feature importance analysis, NH3-H, TN, W-Temp, COD_Mn_, SO_2_, DO, and TP were selected as input variables for pH prediction, TN, pH, TP, EC, DO, and W-Temp were selected as input variables for NH3-N prediction, and TN, W-Temp, NH3-N, A-Temp, TP, and Turb were selected as input variables for DO prediction.

### Predictive sequence decomposition based on CEEMD

**Figure 6:**
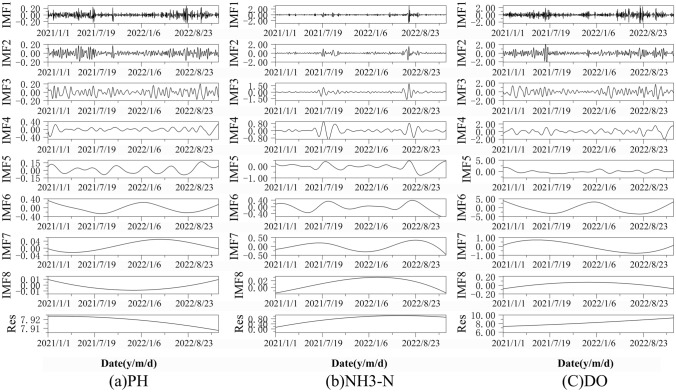
Forecast sequence decomposition of Kangdian station data.

**Figure 7:**
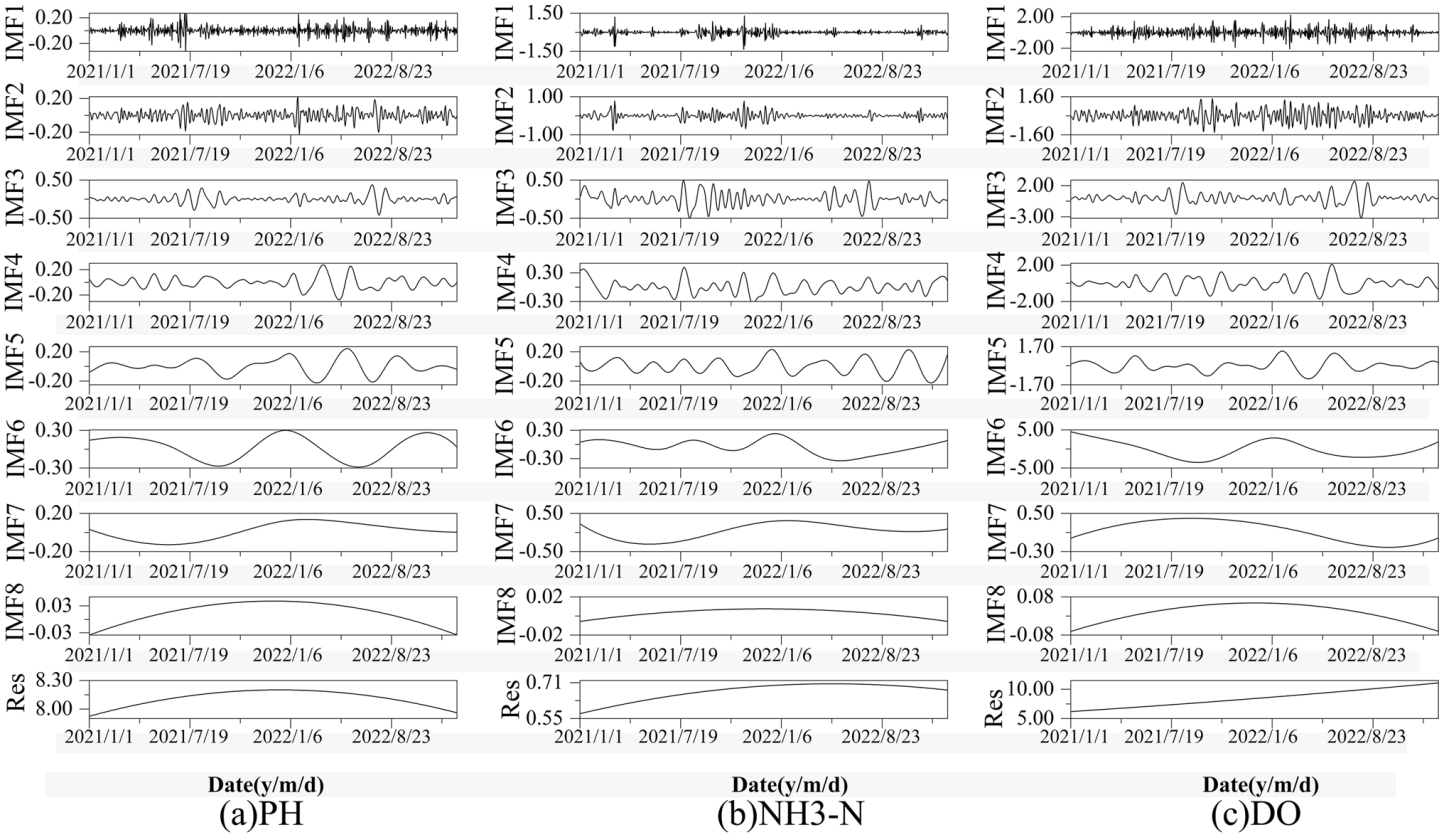
Forecast sequence decomposition of Banqiao station data.

It can be seen from Fig. 5 that there are high-frequency components and noise in the original water pollution prediction sequences, and it may be difficult to fit the model by directly using these sequences for prediction. Each sequence needs to be decomposed before modeling to reduce the impact of high-frequency components and noise on the model performance. Therefore, CEEMD was used to decompose the three prediction sequences of pH, NH3-N and DO at the Kangdian and Banqiao stations. As shown in Figs. 6 and 7, the three prediction sequences of the two stations were decomposed into eight high-frequency components and a residual term, and the signal curve of the CEEMD-decomposed predicted sequence component gradually tended to become stable as the frequency decreased. The fluctuation characteristics of the subsequence from IMF4 to IMF8 gradually became weaker, with an obvious periodic trend. Compared with the original sequence, the decomposed sequence obviously has better stationarity and periodicity.

To evaluate the effect of the CEEMD on the prediction sequences, the index of completion (IC) was selected to compare the CEEMD, EMD and EEMD data differences. The IC is the root mean square error of the decomposition sequence and the original sequence and is often used to compare the differences between the decomposed component sequence data and the original data. As shown in Table 2, EEMD significantly increased the error after reconstruction. The main reason is that EEMD eliminates the noise of the original sequence by adding auxiliary white noise, aiming to overcome the modal aliasing phenomenon existing in EMD. Added white noise is difficult to completely eliminate, resulting in a significant increase in the reconstruction error of the model. CEEMD is used to introduce white noise into each decomposition, ensuring that the error after reconstruction returns to the original order of magnitude. The guarantees accurate subsequent prediction results, effectively solves the modal oscillation phenomenon in EMD and verifies the validity of the method.

**Table 2:** Evaluation index comparisons among EMD, EEMD and CEEMD.

Evaluation index	EMD	EEMD	CEEMD
IC	3.13 × 10^–15^	0.017	3.19 × 10^–15^
IMF number	6	8	8

### RF-CEEMD-LSTM prediction

**Figure 8:**
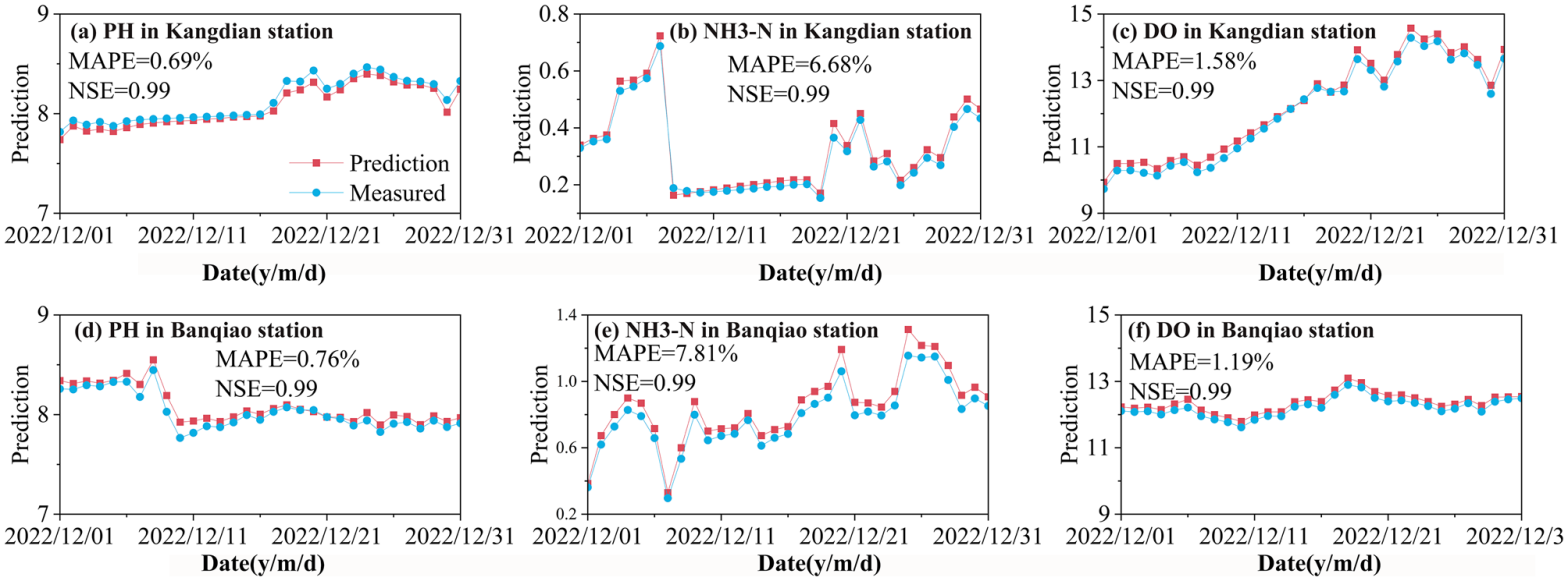
The prediction results of the RF- CEEMD-LSTM model.

The feature selection results of RF analysis were used as the input variables of the LSTM model, and LSTM was used to predict the 8 IMF components and 1 residual item obtained from the CEEMD and finally integrate the prediction sequence to obtain the pH, NH3-N and DO model predictions. The data from January 2021 to November 2022 were used as training samples to train the model and continuously adjust the parameters. After comprehensive consideration and multiple experiments, the network parameters of each water quality index prediction model were determined and are shown in Table 3. The water quality data from December 1st to December 31st, 2022, were input as test samples into the trained model. The results of each water quality index prediction model are shown in Fig. 8. The NSE of the prediction results was 0.99, demonstrating that the RF-CEEMD-LSTM model has a good prediction effect. From the MAPE values of the prediction results, the prediction accuracy of the model for pH (0.69% and 0.76% MAPE) was significantly higher than the prediction accuracies of NH3-N (6.68% and 7.81% MAPE) and DO (1.58% and 1.19% MAPE). The main reason may be that the pH sequence is more stable (Fig. 4), and the RF-CEEMD-LSTM model can easily capture the potential rules of the sequence. Due to the high volatility of the NH3-N sequence, even if the original sequence was decomposed into 8 IMF components and 1 residual term by CEEMD, the decomposed IMF1 and IMF2 components still have certain volatility, which leads to the relatively poor prediction effect of the RF-CEEMD-LSTM model on NH3-N. Nevertheless, the proposed RF-CEEMD-LSTM model has a MAPE of water pollution prediction within 8%. It can predict the trend of water pollution more accurately.

**Table 3:** Network parameters corresponding to each water quality index.

Index	Number of hidden layer neurons	Learning rate	Iterations	Step
pH	20; 40	0.001	300	24
NH3-N	10; 20	0.001	250	24
DO	10; 10	0.003	250	24

### Model comparative analysis

To further analyze the performance of the proposed RF-CEEMD-LSTM model, the differences in water pollution prediction based on the LSTM, RF-LSTM, CEEMD-LSTM and RF-CEEMD-LSTM models were compared. As shown in Table 4, the RF-CEEMD-LSTM prediction results have the smallest deviation from the actual value. The MAPE values of the RF-CEEMD-LSTM model were 0.73% (pH), 7.25% (NH3-N) and 1.39% (DO), and the NSE values of the prediction results were all above 0.99. In contrast, there was a large deviation between the prediction results of the single LSTM model and the actual results. In the prediction of NH3-N, the MAPE and NSE of the LSTM model were 14.58% and 0.88, respectively, which indicates that combined forecasting methods can effectively improve the overall forecasting performance by preprocessing the data through feature selection and decomposition. By comparing the prediction effects of the RF-LSTM and RF-CEEMD-LSTM models, it was found that the RMSE values of RF-LSTM for the three index predictions were 0.101, 0.128 and 0.28, and the RMSE values of the RF-CEEMD-LSTM model were 0.061, 0.071 and 0.18. The RMSE of the RF-CEEMD-LSTM model was 35.7–44.5% lower than that of the RF-LSTM model. Therefore, CEEMD significantly improved the prediction accuracy of the model. By comparing the prediction effects of the CEEMD-LSTM and RF-CEEMD-LSTM models, it was found that the RMSE of the prediction results of the RF-CEEMD-LSTM model was 5.3–22.8% lower than that of CEEMD-LSTM. This result proves that the use of RF for feature selection is effective in improving the model accuracy.

**Table 4:** Comparison of the model prediction accuracy.

Index	Model	NSE	RMSE (mg/L)	MAPE (%)	MAE (mg/L)
pH	LSTM	0.91	0.218	9.31	0.071
	RF-LSTM	0.94	0.101	7.52	0.068
	CEEMD-LSTM	0.975	0.079	3.46	0.061
	RF-CEEMD-LSTM	0.99	0.061	0.73	0.057
NH3-N	LSTM	0.88	0.256	14.58	0.073
	RF-LSTM	0.92	0.128	11.03	0.061
	CEEMD-LSTM	0.95	0.087	8.97	0.049
	RF-CEEMD-LSTM	0.99	0.071	7.25	0.044
DO	LSTM	0.91	0.32	10.21	0.25
	RF-LSTM	0.93	0.28	8.22	0.21
	CEEMD-LSTM	0.97	0.19	4.99	0.18
	RF-CEEMD-LSTM	0.99	0.18	1.39	0.16

## Discussion

In this work, RF was used to analyze the main factors affecting different water quality characteristics, and on this basis, input variables for different prediction indicators were identified. The results of the feature importance analysis indicated that the main factors affecting the prediction of pH are not only water pollution indicators (NH3-N, TN, W-Temp, COD_Mn_, DO, and TP) but also the concentration of SO_2_ in the air. Previous studies have shown that industrial activities are one of the most closely related factors^1^ affecting the water quality of the Huaihe River Basin. The combustion and emission of sulfur containing fuels in industrial activities exacerbate the severity of SO_2_ pollution in the air, and SO_2_ entering the river with rainwater changes the pH balance of the river^39^. In the prevention and control process of river water pollution, it is possible to reduce or block SO_2_ pollution and acidic water quality by increasing the emission standards of industrial waste gas. It was also found that the main factors affecting the change in NH3-N are TN and pH, and the main factors affecting the change in DO in water bodies are temperature and TN. TN has an important impact on pH, NH3-N, and DO in the Huaihe River Basin and is one of the most critical indicators affecting water quality. This discovery is consistent with Feng et al.^40^. The results in Table 4 show that by selecting input variables for different prediction indicators using RF, the RMSE of the model prediction results was reduced by 5.3–22.8%, significantly improving the model prediction accuracy. Therefore, in the construction of water pollution prediction models, the impact of indicator selection on the model performance should be considered^19^. Although this impact is not as significant as the impact of sequence decomposition on the model performance, it is an aspect that cannot be ignored.

Before constructing the water pollution prediction model, three prediction sequences were decomposed into eight IMF components and one residual using CEEMD in this study. By comparing the decomposition effects of EMD, EEMD, and CEEMD, it was found that the CEEMD model not only overcame the error caused by EMD mode aliasing but also significantly reduced the reconstruction error of EEMD by adding complementary white noise sequences, resulting in the best decomposition effect. This is also an important reason why some recent studies on hydrology^41^ and water environment^23^ have recommended the use of CEEMD method for data decomposition.

In order to compare the model performance more fairly, the performance of similar models in water pollution prediction in the latest research was statistically. As shown in Table 5, the proposed RF-CEEMD-LSTM model has the highest NSE, and the lowest MAPE, RMSE, and MAE, indicating that the proposed RF-CEEMD-LSTM has significantly better prediction accuracy than these similar water pollution prediction models. The main reason is that this study used RF algorithm to screen the most suitable input variables before constructing water pollution prediction model, and used CEEMD algorithm to decompose the filtered sequence dataset, the LSTM model can more comprehensively capture the fluctuation characteristics of these sequence data. In the prevention and control of water pollution, the proposed high accuracy water pollution prediction can reflect the current pollution situation of water bodies and the future trend of water body changes. Combining the analysis results of the feature importance, the main factors affecting water pollution can be quantified, which can provide a certain decision-making basis for relevant governance work. The trend perception of water pollution can transform the management of water resource pollution from post treatment to pre prevention and control, has long-term significance for improving the existing water pollution prevention and control situation and promoting the scientific development of the water environment.

**Table 5:** Comparison of prediction accuracy against similar models.

Model	NSE	RMSE (mg/L)	MAPE (%)	MAE (mg/L)	Source
RF-CEEMD-LSTM	0.99	0.061	0.73	0.057	This work
EEMD-LSTM	0.95	0.094	1.47	0.070	Luo et al.^42^
EMD-LSTM	0.94	0.27	2.66	–	Zhang et al.^20^
BPNN	–	0.189	1.70	0.165	Li et al.^43^
LSTM	–	0.085	1.11	0.075	Li et al.^43^

There are still some limitations in this study due to the limited data and research subject. This study uses daily water pollution data to construct water pollution prediction model, which can be used for short-term water pollution prediction and prevention. The applicability of the proposed method for water pollution prediction at other scales still needs further research. Future research can collect longer sequence data to construct weekly and monthly scale water pollution prediction models, which can provide more comprehensive support for water pollution prevention and control. And this study only constructed a prediction model for pH, NH3-N and DO. Future research can explore the effectiveness of the proposed method in predicting other water pollution indicators, especially for heavy metal pollution prediction, which is very useful for water supply safety prevention and control.

## Conclusions

In order to improve the performance of water pollution prediction models and effectively explain the main influencing factors of water pollution. This study proposed a hybrid model for water pollution prediction based on RF-CEEMD-LSTM to predict the changes in pH, NH3-N and DO. A hybrid water pollution prediction model was constructed for the Kangdian and Banqiao stations in the Huaihe River Basin, and the performance of the model was verified using statistical evaluation indicators. Various similar models were used to compare the performance of the constructed model. The main conclusions can be divided into three aspects.

1. The RF algorithm was used to analyze the feature importance of various water pollution prediction variables in this study, and the results showed that TN is the most critical factor affecting water quality changes. Water pollution prevention and policy formulation need pay more attention to TN reduction strategies.
2. The IC value of CEEMD in data decomposition is significantly lower than EMD, and the RMSE of the prediction model using CEEMD (RF-CEEMD-LSTM) is lower than that using EMD (EMD-LSTM) and EEMD (EEMD-LSTM), indicating that using CEEMD can effectively improve the performance of water pollution sequence prediction models. CEEMD can be one of the most effective methods for nonlinear non-stationary data decomposition.
3. The comparisons between different models and the experimental results show that the RMSE value of the proposed RF-CEEMD-LSTM model is 62.6%, 39.9% and 15.5% lower than those of the LSTM, RF-LSTM and CEEMD-LSTM models, indicating that proposed model can provide superior predictive performance. Proposed RF-CEEMD-LSTM model could provide references for improving water pollution prediction method.

## Data Availability

The datasets used during the current study available from the corresponding author on reasonable request.
